# Prevalence and costs of treating uncomplicated stage 1 hypertension in primary care: a cross-sectional analysis

**DOI:** 10.3399/bjgp14X681817

**Published:** 2014-09-29

**Authors:** James P Sheppard, Kate Fletcher, Richard J McManus, Jonathan Mant

**Affiliations:** Nuffield Department of Primary Care Health Sciences, University of Oxford, Oxford.; Primary Care Clinical Sciences, University of Birmingham, Birmingham.; Nuffield Department of Primary Care Health Sciences, University of Oxford, Oxford.; Primary Care Unit, University of Cambridge, Cambridge.

**Keywords:** antihypertensive agents, cardiovascular disease risk, guidelines, primary prevention

## Abstract

**Background:**

Treatment for uncomplicated stage 1 hypertension is recommended in most international guidelines but there is little evidence to indicate that therapy is beneficial.

**Aim:**

To estimate the prevalence of this condition in an untreated population and the potential costs of initiating therapy in such patients.

**Design and setting:**

Cross-sectional study of anonymised patient records in 19 general practices in the West Midlands, UK.

**Method:**

Data relating to patient demographics, existing cardiovascular disease (CVD), and risk factors (blood pressure and cholesterol) were extracted from patient records. Patients with a blood pressure of 140/90–159/99 mmHg, no CVD, and <20% 10-year cardiovascular risk were classified as having uncomplicated stage 1 hypertension. Missing data were imputed. The prevalence of untreated, uncomplicated stage 1 hypertension was estimated using descriptive statistics and extrapolated using national data. The cost of achieving blood pressure control in this population was examined in a cost–impact analysis using published costs from previous studies.

**Results:**

Of the 34 975 patients (aged 40–74 years) in this study, untreated, uncomplicated stage 1 hypertension was present in 2867 individuals (8.2%, 95% confidence interval [CI] = 7.9 to 8.5). This is equivalent to 1 892 519 patients in England and Wales, for whom the additional cost of controlling blood pressure, according to guidelines, was estimated at £106–229 million per annum, depending on the health professional delivering care.

**Conclusion:**

Untreated, uncomplicated stage 1 hypertension is relatively common, affecting 1 in 12 patients aged 40–74 years in primary care. Current international guidelines and pay-for-performance targets, if followed, will incur significant costs for a patient benefit that is debatable.

## INTRODUCTION

Hypertension is a key risk factor for the development of cardiovascular disease (CVD),^1^ the major cause of morbidity and mortality worldwide.^2^ Classifications of hypertension are, by definition, arbitrary and, although most guidelines now include consideration of underlying CVD risk, many recommend pharmacological treatment of blood pressure when it is sustained at ≥140/90 mmHg, regardless of the underlying risk, end organ damage, diabetes, or history of CVD (Table 1).^3^–^13^

**Table 1.** Summary of treatment recommendations for patients with stage 1, 2, and 3 hypertension

**Guideline**	**Country/region**	**Year**	**Low CVD risk**			**High CVD risk^a^**			**Notes**
	
			**Stage 1 HT**	**Stage 2 HT**	**Stage 3 HT**	**Stage 1 HT**	**Stage 2 HT**	**Stage 3 HT**	
European Society of Hypertension^3^	Europe	2013	Therapy	Therapy	Therapy	Therapy	Therapy	Therapy	Initiate therapy in low-risk stage 1 HT if lifestyle modification is unsuccessful
Canadian Hypertension Education Program^4^	Canada	2013	Therapy	Therapy	Therapy	Therapy	Therapy	Therapy	Therapy should only be initiated if mean BP is raised on three occasions (high risk or stage 2–3 HT) or five occasions (stage 1 HT and low risk)
National Institute for Health and Care Excellence^12^	UK	2011	Lifestyle advice	Therapy	Therapy	Therapy	Therapy	Therapy	Diagnosis of hypertension is based on a combination of clinic and ambulatory BP readings
National Heart Foundation of Australia^11^	Australia	2010	Lifestyle advice	Lifestyle advice	Therapy	Therapy	Therapy	Therapy	Treatment is also recommended in patients with moderate CVD risk (10–15%) and raised BP (>140/90 mmHg)
Latin American Society of Hypertension^7^	Latin America	2009	Therapy	Therapy	Therapy	Therapy	Therapy	Therapy	Therapy should only be initiated in patients with stage 1 or 2 HT if raised BP persists for ‘a few weeks’ (in those with moderate CVD risk) or ‘several months’ (in those with low CVD risk)
Japanese Society of Hypertension^9^	Japan	2009	Therapy	Therapy	Therapy	Therapy	Therapy	Therapy	Therapy should be initiated in patients with stage 1 HT if raised BP is sustained after a period of lifestyle modification
American Heart Association^5^,^6^,^13^	US	2006–2014	Therapy	Therapy	Therapy	Therapy	Therapy	Therapy	JNC8 suggests patients aged ≥60 years should only be treated if BP is ≥150/90 mmHg
New Zealand Guidelines Group^10^	New Zealand	2003	Lifestyle advice	Lifestyle advice	Therapy	Therapy	Therapy	Therapy	Treatment is based solely on CVD risk: those with high CVD risk should receive therapy. Stage 3 HT = ≥170/100 mmHg
WHO/International Society of Hypertension^8^	World	2003	Therapy	Therapy	Therapy	Therapy	Therapy	Therapy	

a Includes estimated CVD risk ≥20%, existing CVD, diabetes, renal disease, and target organ damage. Stage 1 hypertension = 140/90–159/99 mmHg; stage 2 hypertension = 160/100–179/109 mmHg; stage 3 hypertension = ≥180/110 mmHg. BP = blood pressure. CVD = cardiovascular disease. HT = hypertension. JNC8 = Eighth Joint National Committee. WHO = World Health Organization.

The treatment of uncomplicated (that is, low risk of CVD) stage 1 hypertension (sustained blood pressure of 140/90–159/99 mmHg) has long been considered controversial due to the paucity of evidence to support such a strategy.^14^–^17^ A recent Cochrane Review^18^ examined 8912 patients from four clinical trials and found no significant reduction in mortality or cardiovascular events with treatment. Despite these data, new international guidelines and pay-for-performance indicators continue to recommend treatment.^3^,^4^,^19^ Only guidelines in the UK, Australia, and New Zealand recommend lifestyle advice rather than pharmacological interventions for uncomplicated stage 1 hypertension (Table 1).^10^–^12^

The impact of these recommendations on clinical practice is unclear. Although the prevalence of hypertension is well documented across the world,^20^–^24^ few studies have examined the prevalence of stage 1 hypertension^25^,^26^ and none, to the authors’ knowledge, have examined its prevalence with regard to CVD risk. This study aimed to establish the total number of individuals potentially requiring treatment for uncomplicated stage 1 hypertension, according to international guidelines, in a typical UK primary care population. These estimates were extrapolated to national data^27^ and the cost impact of achieving blood pressure control was quantified in this population using published costs.^28^,^29^

## METHOD

### Data collection

A cross-sectional retrospective study of anonymised, primary care medical records was conducted in 19 general practices across the West Midlands, UK. The methods of data collection have been detailed elsewhere.^30^,^31^ Briefly, relevant data were extracted from patient medical records using MIQUEST software. All data queries were run between 17 October 2008 and 6 October 2009; extracted data included:

- demographic information;
- cardiovascular risk-factor details; and
- all cholesterol and blood pressure lowering medication prescribed within the 90 days prior to the query date.

**How this fits in**

There are varying recommendations on how to treat people with uncomplicated stage 1 hypertension, but many guidelines recommend therapy for all whose blood pressure is ≥140/90 mmHg, despite the lack of robust clinical trial evidence supporting treatment. At least 1 in 12 patients aged 40–74 years old have the condition but receive no treatment for it. The cost of initiating treatment in these individuals in England and Wales in line with current international guidelines, would be substantial (£106–229 million: US$180–389 million and €134–289 million) in the first year, but the benefits for patients are unknown.

This study focused on individuals aged 40–74 years, as younger people require further assessment for secondary causes of hypertension before treatment is administered^12^ and older individuals are likely to fall into the high CVD risk group and, therefore, not be relevant to the research questions addressed here.

### Definition of uncomplicated stage 1 hypertension

This study aimed to establish the prevalence of untreated, uncomplicated stage 1 hypertension. As such, patients with no blood pressure reading from the preceding 5 years, those with existing CVD, or those already receiving blood pressure or cholesterol-lowering therapy were not considered in this analysis; it was assumed that patients receiving blood pressure- or cholesterol-lowering therapy had been at high risk of CVD prior to initiation.^12^,^32^ The remaining patients formed a potential primary-prevention population and were subdivided on the basis of blood pressure level (<140/90 mmHg = normotension; 140/90–159/99 mmHg = stage 1 hypertension; ≥160/100 mmHg = stage 2–3 hypertension) and CVD risk score.

Given that the classification of hypertension was based on only the most recent clinic blood pressure reading, the prevalence of stage 1 hypertension was adjusted to account for the 56% positive predictive value (PPV) of clinic blood pressure measurements around the diagnostic threshold for hypertension.^33^ The prevalence of stage 2–3 hypertension was not adjusted due to uncertainty about the PPV of blood pressure measurement at higher levels. No attempt was made to impute missing blood pressure data.

### Definition of cardiovascular risk status

Where possible, data on cardiovascular risk factors were used to estimate CVD risk using the Framingham equation.^34^ This risk calculator was recommended by the National Institute for Health and Care Excellence (NICE)^35^ at the time of data collection; it is now recognised, however, that other risk calculators^36^ may be more appropriate for use in UK populations.^32^ CVD risk scores were adjusted by a factor of 1.4 or 1.5 for patients of South Asian origin or a family history of premature cardiovascular events respectively.^35^ Where total or high-density lipoprotein (HDL) cholesterol information was not available, values were imputed using the observed mean cholesterol for patients of the same sex and age group (5-year age bands) with existing cholesterol readings. All other risk factors were assumed not to be present if they were absent from the patient medical records.

### Estimation of costs

The potential costs of treating uncomplicated stage 1 hypertension were examined in a cost–impact analysis using the methodology utilised by NICE for the development of costing tools to assist the implementation of new guidance.^37^ This methodology uses data describing the affected population, the required activity, and the cost of that activity to estimate the financial impact of implementing new guidance on health service budgets. It does not consider these costs in the context of quality-adjusted life years gained, as is the case in a health economic analysis.

The level of new activity generated by guideline implementation was defined in the present study as the number of drugs required to control blood pressure to <140/90 mmHg in each patient and the health professional’s time incurred when prescribing and monitoring the treatment regimen. These figures were estimated using data from the Hypertension Optimal Treatment trial.^38^ Drug choice was guided by the NICE treatment algorithm, which recommends different combinations of therapy according to patient age and ethnic group.^12^

The costs of resource utilisation were estimated using the *NHS Electronic Drug Tariff* 28 (cost of drug prescription) and *Unit Costs of Health and Social Care 2012* 29 (cost of service provision) (Table 2). This analysis focused on the short-term costs of guideline implementation (first year); it did not consider any possible cost savings resulting from future reductions in cardiovascular morbidity or mortality as it has been argued that such reductions would be minimal in this population.^18^

**Table 2.** Unit costs of treatment and NHS service delivery required to achieve blood pressure control in patients with uncomplicated stage 1 hypertension

**Cost type**	**Cost per unit, £**	**Annual cost per patient, £**			
		**Patients requiring no drugs**	**Patients requiring one drug**	**Patients requiring two different drugs**	**Patients requiring three different drugs**
**Drugs^a^**					
Amlodipine 10 mg (28-tablet pack)	1.07 (1 pack)	0	13.96	27.92	41.87
Ramipril 10 mg (28-capsule pack)	1.30 (1 pack)	0	16.96	33.92	50.87
Indapamide 2.5 mg (28-tablet pack)	1.40 (1 pack)	0	18.26	36.53	54.79
Total treatment for a patient aged <55 years^b^	-	0	16.96	30.92	49.18
Total treatment for a patient aged ≥55 years^b^	-	0	13.96	30.92	49.18

**Service delivery^c,d^**					
Practice nurse	45/hour	11.25	22.50	33.75	45.00
Nurse specialist	81/hour	20.25	40.50	60.75	81.00
GP	185/hour	36.00	72.00	108.00	144.00

**Total cost of treatment (aged ≥55 years)**					
Drug + practice nurse	-	11.25	36.46	64.67	94.18
Drug + nurse specialist	-	20.25	54.46	91.67	130.18
Drug + GP	-	36.00	85.96	138.92	193.18

a *Costs taken from* NHS Electronic Drug Tariff *database, correct as of May 2013.^28^*

b As defined by the National Institute for Health and Care Excellence treatment algorithm.

c Costs of service delivery given per consultation (unless otherwise stated) based on a face-to-face, 15-minute nurse consultation or a 11.7-minute GP consultation.

d *Costs taken from* Unit Costs of Health and Social Care 2012*.^29^*

### Analysis

Descriptive statistics were used to define the proportion of patients in each group with different levels of blood pressure control (normotension, stage 1, and stage 2–3 hypertension). Data are presented according to age (40–54 years and 55–74 years) and sex; comparisons were made using χ^2^ tests.

The number of people with untreated, uncomplicated stage 1 hypertension in the population of England and Wales was estimated by extrapolating from the local prevalence estimates derived from this study to national population data.^27^ All data are presented as a mean, ± standard deviation (SD), and proportion of the total population with 95% confidence intervals (CIs) (unless otherwise stated). Costs were calculated in pounds sterling and converted to US dollars and euros for comparison purposes. All analyses were carried out using SPSS software (version 21).

## RESULTS

### Population characteristics

Of the 90 516 patients registered at participating practices, 34 975 were aged 40–74 years and could have been included in this analysis; however, of these, 2550 (7.3%) patients had existing CVD, 7406 (21.2%) were already on treatment, and 4764 (13.6%) had no clinic blood pressure readings in the preceding 5 years from the data extraction date. Of the remaining 20 255 patients (57.9%), 4985 (14.3%) had total and HDL cholesterol information available to calculate CVD risk, leaving 15 270 (43.7%) where cholesterol was imputed. Diabetes, chronic kidney disease, and ischaemic heart disease were the most common comorbidities (Table 3).

**Table 3.** Characteristics of the study population, *n* = 34 975

**Characteristic**	***n* (%)^a^**
Female	16990 (49)
Age, mean (SD)	54 (± 10)
South Asian ethnicity	1899 (12)^b^
Family history of CVD	4213 (12)
Diabetes	2598 (7)
Left ventricular hypertrophy	77 (0)
Chronic kidney disease	1302 (4)
Myocardial infarction	733 (2)
Peripheral vascular disease	487 (1)
Heart failure	253 (1)
Ischaemic heart disease	1678 (5)
Stroke	331 (1)
Transient ischaemic attack	295 (1)

a Unless otherwise stated.

b Percentage of 15 825 patients in whom ethnicity was recorded. Ethnicity is typically collected by self-report at registration; the relatively low proportions recorded reflect patients who have been registered for some years. CVD = cardiovascular disease. SD = standard deviation.

### Prevalence of untreated, uncomplicated stage 1 hypertension

**Figure 1.**
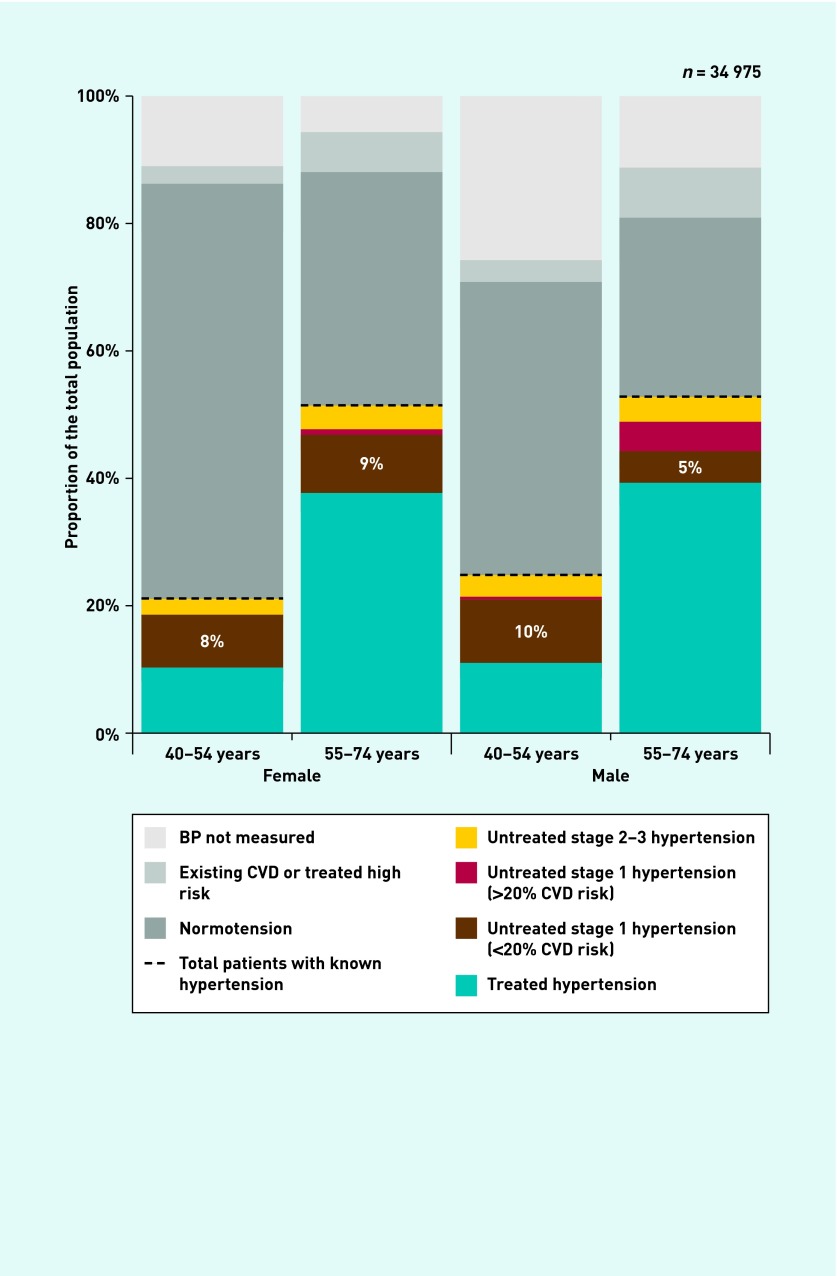
***Prevalence of hypertension by age and sex.*** *Treated hypertension = patients prescribed antihypertensive therapy. Normotension =* <*140/90 mmHg. Untreated stage 1 hypertension = 140/90–159/99 mmHg. Untreated stage 2–3 hypertension = ≥160/100 mmHg. Prevalence of stage 1 hypertension was adjusted by a factor of 0.56 to account for the 56% positive predictive value of clinic BP measurements. All patients with a clinic BP of ≥160/100 mmHg were considered to have stage 2 hypertension due to uncertainty about whether repeated measurement would affect classification at higher BP levels. BP = blood pressure. CVD = cardiovascular disease.*

A total of 12 647 patients had hypertension, of which 4421 patients (35.0%) were not receiving antihypertensive treatment. In patients receiving no treatment, stage 1 hypertension was present in 3321 individuals (9.5%, 95% CI = 9.2 to 9.8), of whom 2867 (8.2%, 95% CI = 7.9 to 8.5) had low CVD risk (that is, ‘uncomplicated’). Untreated, uncomplicated stage 1 hypertension was more common in younger patients (1729 patients, 9.1%, 95% CI = 8.7 to 9.5 [40–54 years] versus 1138 patients, 7.1%, 95% CI = 6.7 to 7.5 [55–74 years], *P*<0.001) and females (1465 patients, 8.6%, 95% CI = 8.2 to 9.1 [females] versus 1402 patients, 7.8%, 95% CI 7.4 to 8.2 [males], *P*=0.005), reflecting an increased risk of CVD in older patients and males. Figure 1 shows the prevalence of hypertension and risk by age and sex. CVD risk is well known to be higher in males and older people.^34^

By applying these figures to national population estimates^27^ for people aged 40–74 years, 1 892 519 patients in England and Wales (approximately 1 in 12 people) were estimated to have untreated, uncomplicated stage 1 hypertension.

### Cost of treating uncomplicated stage 1 hypertension

Based on the cost of a practice nurse measuring a patient’s blood pressure and delivering hypertension management (Table 2), the total cost of treating uncomplicated stage 1 hypertension in the sample population studied here was estimated to be £160 129 per year (Table 4). Extrapolated to the national population of England and Wales, this figure rises to £106 million per year. If hypertension care was delivered by a GP, this figure would rise to £229 million per year (US$180–389 million or €134–289 million) (Table 4).

**Table 4.** Costs of treating uncomplicated stage 1 hypertension across the sample and population of England and Wales, according to the health professional delivering treatment

**Population**		**Cost, £^a^**
Sample population, patients aged 40–74 years	Total, *n*	34 975
	Untreated, uncomplicated stage 1 hypertension,^b^ *n*	2867
	Total cost of treatment if delivered by practice nurse^c^	160 129
	Total cost of treatment if delivered by nurse specialist^c^	228 125
	Total cost of treatment if delivered by GP^d^	347 117

Population of England and Wales, patients aged 40–74 years^e^	Total population, *n*	23 284 217
	Untreated, uncomplicated stage 1 hypertension,^b^ *n*	1 892 519
	Total cost of treatment if delivered by practice nurse^c^	105 619 247
	Total cost of treatment if delivered by nurse specialist^c^	150 500 331
	Total cost of treatment if delivered by GP^d^	229 042 227

a Unless otherwise stated.

b Excludes patients with these conditions for whom blood pressure was not measured within 5 years prior to the study being undertaken.

c Nurse appointment based on 15-minute consultation.

d GP appointment based on 11.7-minute consultation.

e *Population in England and Wales is based on data from the Office for National Statistics, mid-year population estimates (2011).^27^ Drug costs from* NHS Electronic Drug Tariff *database, correct as of May 2013.^28^ Staff costs from* Unit Costs of Health and Social Care 2012*; unit costs based on the cost of face-to-face contact.^29^*

## DISCUSSION

### Summary

This study shows that untreated, uncomplicated stage 1 hypertension is relatively common, affecting 1 in 12 patients aged between 40–74 years in England and Wales. Assuming similar prevalence nationally, it was estimated that approximately 1.9 million people would be affected, with potential annual costs of antihypertensive treatment, if commenced, being £106–229 million (US$180–389million and €134–289 million).

Initiation of therapy in such patients is not recommended (by guidelines) in the UK but many international guidelines do recommend therapy in this group,^3^–^9^,^13^ despite little evidence to suggest it has any benefit.^18^ This study highlights that people with low risk stage 1 hypertension form a significant group for whom more robust evidence of cost-effectiveness is needed prior to commencement of antihypertensive treatment.

### Strengths and limitations

This study used routine data from the West Midlands, UK, and included all registered patients aged 40–74 years from a large cohort of general practices. These data were comparable with the national population aged 35–74 years, in terms of mean age and sex,^27^ prevalence of stroke and ischaemic heart disease, diabetes, untreated hypertension,^22^ and mortality.^39^ It was not possible to compare the characteristics of the study population with national trends in ethnicity and social deprivation, and it is acknowledged that results extrapolated from local to national population data should be interpreted with caution.

Data used here were collected in 2008 and 2009 but the researchers are confident that the prevalence estimates are still relevant today. According to figures from the Quality and Outcomes Framework (QOF),^19^ the prevalence of diagnosed hypertension in England has increased very slightly in the past 5 years (0.3%; from 13.4% to 13.7%). The researchers would not anticipate treatment to have changed very much during this period, given that QOF targets have remained largely the same. Although NICE now recommends the use of ambulatory blood pressure monitoring in the diagnosis of hypertension,^12^ this was not expected to greatly influence the present results as prevalence estimates were adjusted to account for the accuracy (and variability) of clinic blood pressure used in isolation.^33^

The estimates of the prevalence of untreated, uncomplicated stage 1 hypertension presented here may be conservative: patients without a blood pressure reading in the 5 years prior to the study were assumed to not have stage 1 hypertension. Where it was recorded, no correction was made for blood pressure measurement error in patients with stage 2–3 hypertension due to uncertainty about the PPV of readings taken around the 160/100 mmHg threshold. In practice, there is likely to be a degree of measurement error, resulting in some patients with stage 1 hypertension being classed as having stage 2–3 hypertension and vice versa. The Framingham equation may overestimate risk, resulting in the misclassification of individuals (as high risk) and reducing our estimate of the prevalence of uncomplicated stage 1 hypertension;^40^ however, this tool was recommended for use in clinical practice by NICE at the time of data collection.^35^

The assessments of CVD risk undertaken were limited to evaluating existing CVD and CVD risk score; target organ damage, and diabetes were not considered separately, although the latter was included in the Framingham risk equation used here.^34^ Therefore, it is possible that a minority of patients may have been classified as low risk when, in practice, most would be treated as high risk. In the case of diabetes, however, only 211 patients were classified as low risk (1.0%), so the impact of any misclassification is likely to be relatively small. In addition, CVD risk was calculated using cholesterol readings, which were imputed in 15 270 patients. This represents a large proportion of the overall sample (44%) and there remains the possibility that such an approach may have affected the accuracy of risk estimates and, as such, also the overall estimates of untreated, uncomplicated stage 1 hypertension.

Finally, an assumption was made that there are no long-term benefits from the treatment of uncomplicated stage 1 hypertension, in terms of potential cost savings based on the Cochrane Review findings.^18^ Should this assumption be incorrect, these results will underestimate potential benefit.

### Comparison with existing literature

To the authors’ knowledge, this is the first study to document the community prevalence of untreated, uncomplicated stage 1 hypertension anywhere in the world. The prevalence of hypertension in international surveys varies by method of diagnosis, and between countries and continents, from 23.0% to 46.0%^20^–^24^,^41^ (depending on the age range of the population studied); the lowest rates have been reported in Canada^20^ and the highest across Africa.^23^ Estimates of untreated hypertension (12.6%) were comparable with those from previous surveys conducted in Australia (13.1%),^24^ England (15.8%),^22^ and Japan (15.9%).^26^

### Implications for research and practice

This study found that 1 in 12 patients aged 40–74 years have untreated, uncomplicated stage 1 hypertension. The cost of a strategy to treat these patients in clinical practice was estimated to fall between £106 million and £229 million (US$180–389 million or €134–289 million). Whether to initiate treatment in patients with uncomplicated stage 1 hypertension is a source of much debate.^14^–^17^ The recent Cochrane Review by Diao *et al*,^18^ found no evidence that antihypertensive treatment in this population would result in reductions in mortality, coronary artery disease, stroke, or total CVD. Although both the authors of that review and subsequent commentators^15^ point to a lack of power to show benefit, estimates of a number needed to treat (NNT) of 128 for 5 years per cardiovascular event averted suggest few people stand to gain from such treatment.^18^ Indeed, one previous study^42^ suggested that patients judge the maximum acceptable NNT for 5 years to prevent one death in hypertension to be 33 — well below the putative NNT for CVD — rather than mortality benefit from treating uncomplicated stage 1 hypertension.

Since the publication of the Cochrane Review,^18^ three guidelines for the management of hypertension have been published:

- The Canadian Hypertension Education Program guidelines^4^ discounted the findings of the Cochrane Review because of the small sample size and cited a 35-year-old non-placebo trial^43^ as justification for maintaining the recommendation to treat uncomplicated stage 1 hypertension (with the caveat that lifestyle modification may be sufficient to control blood pressure without the initiation of therapy).^4^
- The European Society of Hypertension (ESH) guidelines^3^ also note the lack of power in the Cochrane Review^18^ and highlight the paucity of evidence to guide therapy. However, taking into account the limited available evidence^44^ and the low cost and safety of antihypertensive agents, ESH recommends considering blood pressure-lowering therapy in this population.
- The Eighth Joint National Committee guidelines^13^ revised thresholds for treatment such that patients aged ≥60 years should be given treatment if their blood pressure is ≥150/90 mmHg. In patients aged <60 years, initiation of treatment was encouraged at a threshold of 140/90 mmHg (regardless of risk), although it was acknowledged that this recommendation was based on expert opinion alone.

The data presented here do not suggest that therapy to reduce blood pressure in those with low CVD risk and stage 1 hypertension would provide an adequate cost–benefit ratio, given the high NNT and costs in order to prevent one vascular event, along with the potential adverse consequences an individual may experience having been labelled as having hypertension through side effects and labelling.^18^,^45^,^46^

Untreated, uncomplicated stage 1 hypertension is common, affecting at least 8.2% of the population aged 40–74 years in England and Wales. International guidelines^3^–^9^,^13^ and pay-for-performance targets^19^ encouraging universal thresholds and treatment targets of <140/90 mmHg (regardless of underlying CVD risk) will lead to treatment, with little prospect of benefit at substantial financial cost to healthcare providers across the world. International recommendations should be re-examined to ensure evidence-based treatment until such time as new trials show that benefit outweighs harm and is cost effective.
